# Intravenous immunoglobulin for mortality and inflammatory status in patients with sepsis: a retrospective database study

**DOI:** 10.3389/fimmu.2024.1511481

**Published:** 2025-01-16

**Authors:** Hayabusa Takano, Naoki Kanda, Yuji Wakimoto, Hiroyuki Ohbe, Kensuke Nakamura

**Affiliations:** ^1^ Department of Emergency and Critical Care Medicine, Hitachi General Hospital, Ibaraki, Japan; ^2^ Department of Critical Care and Emergency Medicine, Showa General Hospital, Tokyo, Japan; ^3^ Division of General Internal Medicine, Jichi Medical University, Tochigi, Japan; ^4^ Department of Emergency and Critical Care Medicine, Tohoku University Hospital, Sendai, Japan; ^5^ Department of Critical Care Medicine, Yokohama City University Hospital, Yokohama, Japan

**Keywords:** intravenous immunoglobulin, IVIG, sepsis, PICS, retrospective, propensity score matching

## Abstract

**Background:**

Sepsis is a life-threatening condition caused by severe infection. The efficacy of intravenous immunoglobulin (IVIG) as adjunctive therapy on mortality remains controversial. Moreover, IVIG may favorably affect sepsis-induced immunosuppression like persistent inflammation, immunosuppression, and catabolism syndrome (PICS).

**Methods:**

This study was a retrospective cohort study using inpatient claims database provided by Medical Data Vision, which included approximately 190,000 episodes of intensive care unit admissions in Japanese acute care hospitals between April 2008 and September 2021. We used a propensity score-matched analysis to compare outcomes between the IVIG and control groups. Primary outcomes were 28-day mortality, while secondary outcomes included in-hospital mortality, the Barthel Index at discharge, length of hospital stay and laboratory data (albumin, C-reactive protein (CRP), and lymphocyte count) on days 14 and 28.

**Results:**

Of the 17,626 patients enrolled, 15,159 (786 in the IVIG group and 14,373 in the control group) were included in the analysis. Propensity score matching generated 758 matched pairs. Before matching, 28-day mortality and in-hospital mortality were lower in the control group; however, in the matched cohort, 28-day mortality was significantly lower in the IVIG group than in the control group (90/758 [11.9%] vs 124/758 [16.4%]; risk difference [95% confidence intervals (CI)], -4.5% [-8.0% to -1.0%]; P = 0.015). In-hospital mortality in the matched cohort was also significantly more favorable in the IVIG group (137/758 [18.1%] vs 177/758 [23.4%]; risk difference [95%CI], -5.3% [-9.3% to -1.2%]; P = 0.013). Favorable outcomes in terms of albumin on days14 and 28 and CRP levels on day 28 were observed in the IVIG group.

**Conclusions:**

The administration of IVIG was associated with a reduction in sepsis mortality and favorable outcomes in laboratory parameters and the functional status. These results will contribute to the ongoing debate on the efficacy of IVIG for sepsis. The results obtained herein suggest the benefit of IVIG, particularly in mitigating PICS. Further research, including prospective studies, is warranted to confirm these results and examine long-term outcomes.

## Background

Sepsis is a life-threatening condition caused by severe infection (1). Various adjunctive therapies have been employed for its treatment, including intravenous immunoglobulin (IVIG) (2, 3). IVIG has been reported to exert beneficial effects in patients with sepsis by enhancing phagocytosis through specific antibody supplementation, as well as by inhibiting complement activity, neutralizing harmful toxins, and modulating inflammation (4–7).

However, the effect of IVIG on mortality in patients with sepsis remains controversial in clinical trials (8, 9). A relatively large randomized controlled trial reported that IVIG administration did not improve 28-day mortality in patients with severe sepsis (8). Conversely, a meta-analysis of clinical trials demonstrated lower mortality rates in IVIG-treated patients (9). Nevertheless, many of these studies are outdated, making it challenging to draw definitive conclusions (3). Furthermore, advances in sepsis treatment may have influenced the role of IVIG. Thus, it is necessary to investigate whether IVIG affects mortality in sepsis using contemporary real-world data.

Furthermore, immunomodulatory interventions like IVIG may influence immune status and long-term outcomes in survivors beyond mortality. Prolonged inflammation and associated immunosuppression after acute phase are long-term relevant issue, known as persistent inflammation, immunosuppression, and catabolism syndrome (PICS) (10–12). While inflammation from underlying diseases triggers PICS, immunosuppression becomes its core pathology, increasing vulnerability to secondary and nosocomial infections (13, 14). IVIG may positively impact sepsis-induced immunosuppression, such as PICS, by enhancing immunity through antibody supplementation and exerting anti-inflammatory effects (15). These anti-inflammatory properties have been demonstrated in various diseases (16), and IVIG has been reported to prevent immune anergy by reducing neutrophil dysfunction and preserving lymphocyte function (5). Thus, IVIG administration during the acute phase of sepsis may help to mitigate PICS in survivors.

Herein, to examine the effects of IVIG, we analyzed data from patients with sepsis admitted to intensive care units (ICUs) in Japanese acute care hospitals. We hypothesized that IVIG could reduce mortality and suppress the development of PICS in survivors. Using propensity score, we investigated the effect of IVIG on mortality as the primary outcome. We also evaluated other outcomes including PICS-related biomarkers (17, 18). Sepsis with low immunoglobulin G (IgG) levels has been reported to have higher mortality rates (19, 20), and immunoglobulin supplementation through IVIG might be more effective in such patients, therefore, we conducted subgroup analyses focusing on patients with suspected low IgG levels.

## Materials and methods

### Data source

The administrative claims database of inpatients and laboratory test results in Japan, supplied by Medical Data Vision Co., Ltd. (Tokyo, Japan), was used in this retrospective cohort study. Approximately 190,000 episodes of ICU admissions in Japanese acute care hospitals between April 2008 and September 2021 are included in our database. Administrative data were consistent with the Diagnosis Procedure Combination (DPC) payment system (21).

The Ethics Committee of Hitachi General Hospital gave its approval for this study, which was performed in compliance with the Declaration of Helsinki (2020-131). Since it used anonymized data and had a retrospective design, informed consent was not required.

### Study population

We identified adult (≥18 years) patients who were diagnosed with sepsis and were in the ICU on the day of hospitalization and the following day between April 2008 and September 2021. Patients who developed sepsis during hospitalization (hospital-acquired sepsis) were not included, but patients transferred to the ICU after developing sepsis in another hospital were included. The diagnostic codes of sepsis and the source of infection were based on a previous study using the DPC database (22).

We conducted a landmark analysis to account for an immortal time bias, using the time point of the third day of hospitalization (referred to as “day 2” because the first day of hospitalization was defined as day 0 in the present study). The following patients were excluded from the analysis: (1) pregnant women, (2) patients who were discharged on day 0, 1, or 2, and (3) patients who died by day 2. If a patient was hospitalized more than once, only the first episode was included in the analysis.

Patients administered IVIG during the first three days after hospitalization (day 0, 1, or 2) were assigned to the IVIG group, and those who did not have the code of IVIG during the first three days were assigned to the control group.

### Covariates

We extracted the following data as covariates from our database: age, sex, body mass index (categorized as <18.5, 18.5–25.0, 25.0–30.0, or >30.0 kg/m^2^), smoking status (non-smoker or current/ex-smoker), ambulance use, emergent admission, the Charlson Comorbidity Index defined by International Statistical Classification of Diseases-10 codes (23) (categorized as 0–1, 2–3, or ≥4 points), surgery under general anesthesia (emergent or elective), source of infection, the sequential organ failure assessment (SOFA) score of each organ on day 1, the total SOFA score on day 1, the catecholamine index on day 1, mechanical ventilation, renal replacement therapy (RRT, continuous or intermittent), extracorporeal membrane oxygenation (ECMO), systemic steroid administration and transfusion therapy (packed red blood cells, fresh frozen plasma, platelet concentrates, and albumin preparation) on days 0-1, and the worst values for laboratory data between days 0-1 (highest white blood cell count and C-reactive protein (CRP), lowest lymphocyte count, albumin (Alb), hemoglobin (Hb), and platelet count). We also included ICU type as a covariate. We defined patients who stayed in units with a nurse-to-patient ratio of 1:2 as ICU patients, while those in units with other ratios were classified as high-dependency care unit (HDU) patients.

Since our database did not include information on oxygenation (PaO_2_ and FiO_2_), we defined the respiratory component of SOFA as follows: patients who did not receive oxygen therapy as 0, patients who received oxygen therapy as 1, patients who received non-invasive positive pressure ventilation or a high-flow nasal canula as 2, patients with mechanical ventilation as 3, and patients who received ECMO therapy as 4. In addition, our database did not include information on vital signs or time-specific records of treatments. Therefore, the dose of catecholamines was calculated using prescription records on day 1, and we defined the cardiovascular component of the SOFA score of patients who did not receive catecholamines as 0. The catecholamine dosage was converted to gamma (µg per kg per minute) units, and the total dosage of dopamine, dobutamine, and noradrenaline ×100 and adrenaline ×100 was used as the catecholamine index. In our database, the level of consciousness on admission was recorded using the Japan Coma Scale (JCS); therefore, we calculated the neurological component score using the conversion rule from JCS to Glasgow Coma Scale (24) as follows: a JCS score of 0 or 1 at admission was assigned 0 points; 2 or 3 was assigned 1 point; 10 or 20 was assigned 2 points; 30, 100, or 200 was assigned 3 points; and 300 was assigned 4 points.

### Outcomes

The primary outcome of interest was 28-day mortality. Secondary outcomes were in-hospital mortality, the Barthel index at discharge, length of hospital stay and laboratory data (Alb, CRP, and lymphocyte count) on days 14 and 28. The Barthel index of deceased patients was regarded as 0 (25). Laboratory data on day 14 (day 28) were referred to the nearest day to day 14 within days 11–17 (day 28 within days 25–31). If laboratory data were available on days 13 and 15, data on day 15 were used.

### Subgroup analysis

We conducted a subgroup analysis limited to patients with septic shock, defined by receiving catecholamines during days 0-1. In addition, we performed a subgroup analysis to investigate whether the effects of IVIG on the mortality of patients with sepsis varied with the level of IgG at admission, using the difference between total protein (TP) and Alb as a surrogate marker of IgG. There is currently no well-known consensus on the clinical cut-off value for low IgG; therefore, we divided patients into a low IgG group and high IgG group based on the median value of the difference between TP and Alb.

### Statistical analysis

A propensity score-matched analysis was used to compare outcomes between the IVIG and control groups based on propensity scores for each patient (26, 27). Missing values were imputed using chained equations. To calculate the propensity score for receiving IVIG, a generalized linear regression model with logistic regression using all covariates was employed. The C-statistic was calculated to evaluate the performance of the discrimination of propensity scores. We conducted one-to-one nearest-neighbor matching without replacement using a caliper width set at 20% of the standard deviation for propensity scores. The absolute standardized mean difference (ASD) was used to characterize variations in covariates between the IVIG and control groups. ASDs less than 0.100 were regarded as a negligible imbalance between the two groups. The null hypothesis was assigned and risk differences and the 95% confidence intervals (CIs) of each binomial outcome were calculated. The Barthel index at discharge and laboratory values, which are continuous outcomes, were compared using the Wilcoxon rank-sum test.

A sensitivity analysis was conducted to confirm the robustness of our results using a propensity score weighting method. We performed an overlap weighting analysis because the inverse probability weighting method was unable to achieve a satisfactory level of variable balance (data not shown) (28–30). Overlap weighting is a propensity score weighting method that emphasizes the target population with the greatest overlap in the observed characteristics between the treated and control groups. When we used this method, patients in the treatment (IVIG) group were weighted by the possibility of not administering IVIG (1 – propensity score). On the other hand, patients in the control group were weighted by the possibility of administering IVIG (propensity score). Since weights are limited to ranges between 0 and 1, extreme weights are impossible and truncation is not necessary. Additionally, overlap weighting delivers an exact balance on the mean of each measured covariate when the propensity score is estimated using a logistic regression. Every P-value was two-tailed, with P-values <0.05 being regarded as significant. R (version 4.2.1, R Foundation for Statistical Computing, Vienna, Austria) was used for all statistical analyses. The imputation method and propensity score matching and weighting were performed using the “mice”, “MatchIt”, and “PSweight” packages in that order.

## Results

**Figure 1:**
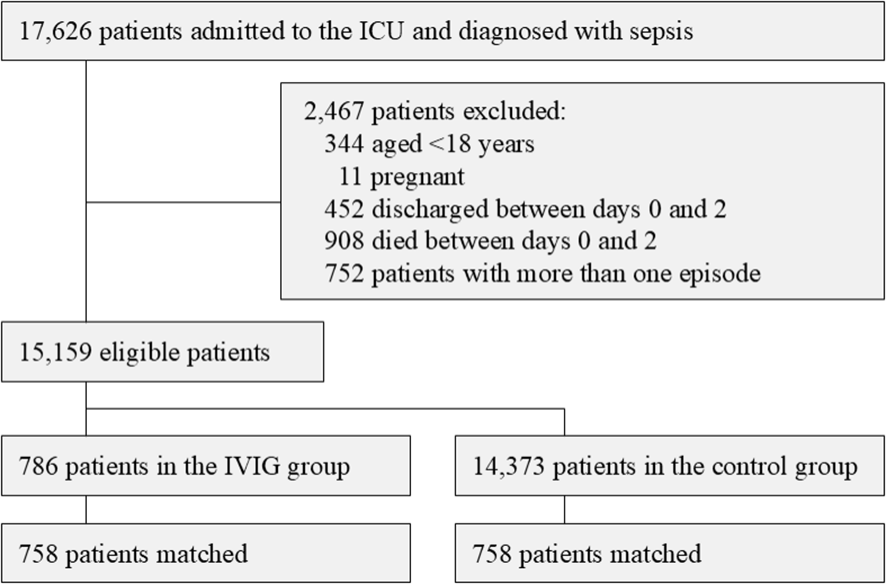
Patient flow chart. ICU, intensive care unit; IVIG, intravenous immunoglobulin.

A total of 17,626 patients were enrolled in this study. After 2,467 patients were excluded, 15,159 eligible patients (786 in the IVIG group and 14,373 in the control group) were included in the propensity score analysis (**Figure 1**).

**Table 1** shows patient characteristics before and after propensity score matching. Before matching, patients in the IVIG group were more likely to be severely ill than patients in the control group.

**Table 1:** Patient characteristics before and after matching.

	Pre-matched cohort			Matched cohort		
Category	IVIG (n = 786)	Control (n = 14,373)	ASD	IVIG (n = 758)	Control (n = 758)	ASD
Age, years old, mean (SD)	72.33 (13.39)	73.47 (15.59)	0.078	72.38 (13.38)	72.18 (14.40)	0.015
Female, n (%)	348 (44.3)	5,868 (40.8)	0.07	330 (43.5)	332 (43.8)	0.005
Body mass index, n (%)						
<18.5	126 (16.0)	3,069 (21.4)	0.137	121 (16.0)	113 (14.9)	0.029
≥18.5, <25.0	488 (62.1)	8,092 (56.3)	0.118	468 (61.7)	483 (63.7)	0.041
≥25.0, <30.0	138 (17.6)	2,472 (17.2)	0.009	135 (17.8)	133 (17.5)	0.007
>30.0	34 (4.3)	740 (5.1)	0.039	34 (4.5)	29 (3.8)	0.033
Current/ex-smoker, n (%)	302 (38.4)	5,629 (39.2)	0.015	291 (38.4)	291 (38.4)	<0.001
Charlson Comorbidity Index, n (%)						
0–1	408 (51.9)	7,386 (51.4)	0.01	392 (51.7)	392 (51.7)	<0.001
2–3	237 (30.2)	4,387 (30.5)	0.008	229 (30.2)	232 (30.6)	0.009
≥4	141 (17.9)	2,600 (18.1)	0.004	137 (18.1)	134 (17.7)	0.01
Ambulance use, n (%)	510 (64.9)	8,605 (59.9)	0.104	489 (64.5)	488 (64.4)	0.003
Emergent admission, n (%)	778 (99.0)	13,392 (93.2)	0.303	750 (98.9)	754 (99.5)	0.06
ICU type, n (%)						
ICU	506 (64.4)	4,171 (29.0)	0.758	479 (63.2)	493 (65.0)	0.039
High-dependency care unit	280 (35.6)	10,202 (71.0)	0.758	279 (36.8)	265 (35.0)	0.039
Surgery under general anesthesia, n (%)	342 (43.5)	4,201 (29.2)	0.3	317 (41.8)	304 (40.1)	0.035
Source of infection, n (%)						
Abdominal	382 (48.6)	5,534 (38.5)	0.205	357 (47.1)	346 (45.6)	0.029
Blood	6 (0.8)	38 (0.3)	0.07	6 (0.8)	5 (0.7)	0.016
Bone and soft tissue	32 (4.1)	538 (3.7)	0.017	31 (4.1)	36 (4.7)	0.032
Cardiovascular	13 (1.7)	490 (3.4)	0.112	13 (1.7)	16 (2.1)	0.029
Central nervous system	16 (2.0)	434 (3.0)	0.063	16 (2.1)	15 (2.0)	0.009
Respiratory	92 (11.7)	4,433 (30.8)	0.481	92 (12.1)	99 (13.1)	0.028
Urogenital	14 (1.8)	863 (6.0)	0.22	14 (1.8)	7 (0.9)	0.079
Others	400 (50.9)	3,009 (20.9)	0.657	373 (49.2)	381 (50.3)	0.021
Total SOFA score, mean (SD)	7.02 (4.18)	3.78 (3.31)	0.859	6.85 (4.12)	7.05 (4.50)	0.046
SOFA, respiratory	1.59 (1.20)	1.07 (1.02)	0.465	1.56 (1.20)	1.67 (1.21)	0.086
SOFA, coagulation	1.22 (1.20)	0.62 (0.92)	0.555	1.19 (1.18)	1.22 (1.18)	0.03
SOFA, liver	0.39 (0.75)	0.35 (0.69)	0.056	0.40 (0.76)	0.43 (0.78)	0.033
SOFA, renal	1.51 (1.72)	0.63 (1.17)	0.599	1.45 (1.69)	1.41 (1.71)	0.026
SOFA, conscious	0.47 (0.98)	0.45 (0.96)	0.02	0.47 (0.97)	0.52 (1.04)	0.048
SOFA, circulation	1.84 (1.79)	0.65 (1.34)	0.753	1.78 (1.78)	1.80 (1.79)	0.015
Catecholamine index, mean (SD)	9.64 (14.92)	2.70 (8.39)	0.574	9.07 (14.47)	9.75 (15.64)	0.045
Mechanical ventilation, n (%)	301 (38.3)	2,461 (17.1)	0.487	280 (36.9)	311 (41.0)	0.084
Extracorporeal membrane oxygenation, n (%)	7 (0.9)	89 (0.6)	0.031	7 (0.9)	11 (1.5)	0.049
Renal replacement therapy, n (%)						
Continuous	186 (23.7)	447 (3.1)	0.633	167 (22.0)	162 (21.4)	0.016
Intermittent	40 (5.1)	399 (2.8)	0.119	37 (4.9)	37 (4.9)	<0.001
Systemic steroid, n (%)	205 (26.1)	2,715 (18.9)	0.173	194 (25.6)	211 (27.8)	0.051
Transfusion therapy, n (%)						
Red blood cells	193 (24.6)	1,104 (7.7)	0.471	174 (23.0)	171 (22.6)	0.009
Fresh frozen plasma	185 (23.5)	495 (3.4)	0.615	160 (21.1)	151 (19.9)	0.029
Platelet concentrate	76 (9.7)	238 (1.7)	0.352	70 (9.2)	64 (8.4)	0.028
Albumin preparation	337 (42.9)	1,259 (8.8)	0.847	310 (40.9)	296 (39.1)	0.038
Laboratory data, mean (SD)						
White blood cell count, 10^9^/L	9,180 (7,550)	10,470 (7,050)	0.177	9,330 (7,570)	9,460 (6,500)	0.019
Lymphocyte count, 10^9^/L	650 (600)	880 (1380)	0.223	650 (600)	670 (580)	0.034
Hemoglobin, g/dL	10.43 (2.22)	11.22 (2.34)	0.349	10.45 (2.22)	10.40 (2.38)	0.022
Platelet count, 10^9^/L	13.88 (9.87)	18.36 (9.87)	0.454	14.06 (9.87)	13.85 (9.57)	0.022
Albumin, g/dL	2.39 (0.63)	2.86 (0.73)	0.69	2.41 (0.61)	2.38 (0.67)	0.038
C-reactive protein, mg/dL	13.42 (10.83)	10.10 (9.72)	0.322	13.47 (10.80)	13.72 (10.98)	0.023

IVIG, intravenous immunoglobulin; ASD, absolute standardized mean difference; SD, standard deviation; ICU, intensive care unit; SOFA, sequential organ failure assessment.

Patients in the IVIG group had worse SOFA scores and a higher percentage were receiving several treatments, such as mechanical ventilation, continuous RRT, catecholamines, systemic steroids, and transfusion therapy. Among 15,159 eligible patients, 5,916 (39.0%) had a diagnosis of abdominal infection, 4,525 (29.9%) of respiratory infection, and 877 (5.8%) of urogenital infection. Propensity score matching generated 758 matched pairs. The median (interquartile range, IQR) duration of IVIG in the IVIG group during hospitalization was 3 (3–3) days, and the median (IQR) total dose was 15 g (12.5 g–15 g). Among the IVIG group, 434 (57.3%) patients received 5 g/day of IVIG for three days, which was a standard regimen in Japan. After matching, 479 (63.2%) patients and 493 (65.0%) patients admitted to the ICU in the IVIG group and in the control group, respectively. Among patients who admitted to the HDU, 271 (97.1%) patients and 263 (99.2%) patients admitted to units with a nurse-to-patient ratio of 1:4, while the remaining patients admitted to 1:5 units. All covariates showed a good balance between the two groups (ASDs of all covariates <0.100). The C-statistic (95%CI) for predicting the administration of IVIG was 0.85 (0.83–0.86). **Supplementary Table 1** shows patient characteristics before the imputation and the percentages of missing values for covariates.

**Table 2** shows the results of primary and secondary outcomes, both before and after matching. Before matching, mortality was lower in the control group; however, in the matched cohort, 28-day mortality was significantly lower in the IVIG group than in the control group (90/758 [11.9%] vs 124/758 [16.4%]; risk difference [95% confidence intervals (CI)], -4.5% [-8.0% to -1.0%]; P = 0.015). In-hospital mortality in the matched cohort was also significantly more favorable in the IVIG group (137/758 [18.1%] vs 177/758 [23.4%]; risk difference [95%CI], -5.3% [-9.3% to -1.2%]; P = 0.013). Length of hospital stay was significantly longer in the IVIG group. Lymphocyte counts on days 14 and 28 did not significantly differ between the two groups; however, favorable outcomes in terms of albumin on days14 and 28 and CRP levels on day 28 were observed in the IVIG group. Changes in Alb, CRP, and lymphocyte counts from admission to day 28 are shown in **Supplementary Figure 1**.

**Table 2:** Outcomes in the pre-matched cohort and propensity score-matched cohort.

	Pre-matched cohort		Matched cohort			
Outcomes	IVIG (n = 786)	Control (n = 14,373)	IVIG (n = 758)	Control (n = 758)	Absolute risk difference (95%CI)	*P* value
Primary outcome						
28-day mortality, n (%)	94 (12.0)	1,273 (8.9)	90 (11.9)	124 (16.4)	-4.5 (-8.0 to -1.0)	0.015
Secondary outcomes						
In-hospital mortality, n (%)	143 (18.2)	1,800 (12.5)	137 (18.1)	177 (23.4)	-5.3 (-9.3 to -1.2)	0.013
The Barthel index at discharge^†^	55 (0–100)	70 (0–100)	55 (0–100)	45 (0–100)	–	0.073
Length of hospital stay, day^††^	34 (21– 54)	16 (9– 30)	33 (21– 53)	25 (15– 51)	–	<0.001
Lymphocyte count on day 14, 10^9^/L	1,040 (680–1,430)	1,130 (780–1,560)	1,050 (700–1,440)	1,090 (730–1,500)	–	0.37
Albumin on day 14, g/dL	2.4 (2.0–2.9)	2.5 (2.1–3.0)	2.5 (2.1–2.9)	2.3 (1.8–2.8)	–	<0.001
C-reactive protein on day 14, mg/dL	3.0 (1.0–7.8)	2.1 (0.7–5.6)	2.9 (1.0–7.6)	3.3 (1.1–8.0)	–	0.144
Lymphocyte count on day 28, 10^9^/L	1,170 (780–1,580)	1,200 (830–1,620)	1,170 (790–1,580)	1,250 (840–1,660)	–	0.562
Albumin on day 28, g/dL	2.6 (2.1–3.0)	2.5 (2.1–2.9)	2.6 (2.1–3.0)	2.2 (1.8–2.7)	–	<0.001
C-reactive protein on day 28, mg/dL	1.7 (0.4–5.0)	1.9 (0.6–5.1)	1.6 (0.4–4.9)	2.2 (0.9–5.6)	–	0.01

IVIG, intravenous immunoglobulin; CI, confidence interval.

Data are represented as medians (interquartile range) unless otherwise indicated.

^†^The scores of patients who died during hospitalization were zero.

^††^Patients who died during hospitalization were excluded.

**Table 3** presents the results of the subgroup analysis restricted to patients with septic shock. In this subgroup, the IVIG group exhibited significantly lower 28-day mortality, with other outcomes showing trends similar to those observed in the primary analysis.

**Table 3:** Outcomes among matched patients with septic shock.

	Patients with septic shock			
Outcomes	IVIG (n = 392)	Control (n = 396)	Absolute risk difference (95%CI)	*P* value
Primary outcome				
28-day mortality, n (%)	66 (16.8)	94 (23.7)	-6.9 (-12.5 to -1.3)	0.02
Secondary outcomes				
In-hospital mortality, n (%)	91 (23.2)	129 (32.6)	-9.4 (-15.6 to -3.1)	0.004
The Barthel index at discharge^†^	32.5 (0–100)	10 (0–90)	–	0.002
Length of hospital stay, day^††^	31 (18– 54)	27 (15– 55)	–	0.067
Lymphocyte count on day 14, 10^9^/L	990 (650–1,360)	1,070 (710–1,420)	–	0.188
Albumin on day 14, g/dL	2.4 (2.0–2.7)	2.0 (1.7–2.6)	–	<0.001
C-reactive protein on day 14, mg/dL	3.8 (1.4–8.7)	4.4 (1.7–10.0)	–	0.135
Lymphocyte count on day 28, 10^9^/L	1,180 (740–1,700)	1,250 (810–1,670)	–	0.666
Albumin on day 28, g/dL	2.4 (2.0–2.8)	2.1 (1.8–2.7)	–	0.001
C-reactive protein on day 28, mg/dL	2.3 (0.7–5.5)	2.4 (1.0–5.3)	–	0.475

IVIG, intravenous immunoglobulin; CI, confidence interval.

Data are represented as medians (interquartile range) unless otherwise indicated.

^†^The scores of patients who died during hospitalization were zero.

^††^Patients who died during hospitalization were excluded.

Since the median value of the difference between TP and Alb on admission was 2.7 g/dL, we defined patients with a value of 2.7 g/dL or less as the low IgG group. The administration of IVIG reduced the risk of 28-day mortality by -6.2% (95%CI, -11.3% to -1.2%) in the low IgG group and by -2.8% (95%CI, -7.7% to 2.0%) in the high IgG group. In-hospital mortality was reduced by -8.4% (95%CI, -14.2% to -2.6%) in the low IgG group and by -2.2% (95%CI, -8.0% to 3.5%) in the high IgG group.

The sensitivity analysis using overlap weighting showed similar results to the main analysis. The rates of 28-day mortality and in-hospital mortality were lower in the IVIG group than in the control group. Absolute risk differences were -2.4% (-4.8% to 0.0%) and -2.5% (-5.3% to -0.3%), respectively (**Supplementary Table 2**).

## Discussion

The present study showed that the administration of IVIG reduced mortality in patients with sepsis. Furthermore, Alb on day14/28 and CRP on day28 as PICS-related biomarkers were significantly favorable in the IVIG group. Length of hospital stay was significantly longer in the IVIG group. The IVIG group showed a trend toward better Barthel Index scores without statistical significance. These effects showed similar trends even when the analysis was restricted to patients with septic shock.

The efficacy of IVIG against mortality in patients with sepsis remains a subject of controversy (8, 9). While our study is retrospective, it contributes to the longstanding debate by demonstrating a potential improvement in prognosis associated with the administration of IVIG. In the pre-matched cohort, the IVIG group exhibited higher disease severity and mortality. After matching, mortality rates were reversed, with the IVIG group showing lower mortality. IVIG contains antibodies against various pathogenic microorganisms and toxins, and has anti-inflammatory properties and actions against various immune cells (4–6), which may have contributed to the reduction in mortality in patients with sepsis.

On the other hand, an observational study, also from Japan, showed that IVIG did not reduce mortality in patients with sepsis (31). This study was a *post hoc* analysis of the JSEPTIC-DIC study (31). They performed propensity score matching with a logistic regression model adjusted for other treatment interventions using generalized estimating equations and showed no significant difference in ICU/in-hospital death between the IVIG and non-IVIG groups (ICU death 21.0% vs 18.1%, in-hospital death 32.9% vs 28.6%) (29). Since there was no information on the timing of adjuvant therapies, such as steroids, RRT, ECMO, and blood transfusion, these treatments were not adjusted for in their study. We used these therapies as covariates to calculate propensity scores, which may have aligned severity among the two groups after matching.

In the present study, Alb levels were slightly higher and CRP levels were lower in the IVIG group. Since Alb and CRP levels and lymphocyte counts are useful for defining the development of PICS (17), the administration of IVIG may be able to control PICS. IVIG preferentially phagocytoses pathogens by opsonization if they contain specific antibodies against the pathogen, and has the potential to cure septic pathology before it enters a spiral of inflammation and anti-inflammation (15, 16). The development of PICS is accompanied by increases in CRP, interleukin (IL)-6, and IL-8 levels. IgG preparations possess anti-inflammatory properties because they contain antibodies against various inflammatory cytokines (5, 6). IVIG also increases the production of the IL-1 receptor antagonist, an anti-inflammatory cytokine (32), which may reduce the development of persistent inflammation. In addition, persistent immunosuppression via bone marrow-derived suppressor cells has been associated with the development of PICS (17). IVIG may prevent delayed immune anergy by attenuating neutrophil dysfunction and directly activating B cell proliferation (5), thereby avoiding sepsis-induced immunosuppression (immune-exhaustion) (15, 16), which may prevent PICS. These mechanisms are possible even without specific antibodies (7).

In addition, the Barthel Index at discharge tended to be higher in the IVIG group without statistical significance. IVIG may reduce disability and maintain good activities of daily living in patients hospitalized with sepsis. In PICS, not only persistent inflammation and immunosuppression, but also hypercatabolism occur, resulting in muscle atrophy and weakness (12). Based on the present results, IVIG has the potential to break this cycle. IVIG may improve the prognosis and quality of life of patients with sepsis not only in the acute phase, but also in the remote phase.

The length of hospital stay was significantly longer in the IVIG group. We considered that the lower mortality in the IVIG group contributed to this result, as more patients survived and required prolonged care.

Previous studies suggested that the prognosis of sepsis may be worse in the low IgG group (19, 20). We performed a subgroup analysis using the difference between TP and Alb, which represents IgG and other globulins in general, as the surrogate marker of serum IgG. The results showed that mortality among patients with sepsis was significantly lower in the low IgG group. Serum IgG levels are low in septic patients from early onset due to the suppression of IgG production, leakage, and muscle wasting (33). Since the immunological effects of IgG may be more attenuated in the low IgG group, the administration of IVIG may increase blood IgG levels and potentially be more effective and beneficial in this group.

The present study has several limitations. There may have been unmeasured and/or unknown confounding factors, and thus the severity might not have been fully adjusted between two groups. We adjusted general anesthesia on day 0–1 as the procedure for source control, however, less invasive source control procedures were not adjusted. Using International Statistical Classification of Diseases-10 codes to identify patients with sepsis has limitations in discrimination. In addition, our database does not contain facility information or any identifier to discriminate between facilities; therefore, our analysis did not consider hospital-level effects. Indeed, in the matched cohort, antibiotics for methicillin-resistant Staphylococcus aureus were administered to 74 (9.7%) patients in the IVIG group and 129 (16.9%) patients in the control group during day 0–1, whereas anti-Pseudomonal agents were administered to 692 (90.7%) and 539 (70.6%) patients, respectively. The difference in broad-spectrum antibiotic use may suggest variations in the quality of intensive care and treatment practices between groups. Furthermore, we did not examine the relationship between the usage (dose and duration) of IVIG administration and the effect on clinical outcomes, therefore, the optimal dosage and duration of IVIG remained unclear.

## Conclusions

The present study showed that IVIG was associated with reduced mortality in patients with sepsis. Furthermore, favorable CRP and Alb levels were observed in the IVIG group, suggesting a potential impact on PICS.

## Data Availability

Publicly available datasets were analyzed in this study. This data can be found here: The administrative claims database of inpatients and laboratory test results in Japan, supplied by Medical Data Vision Co., Ltd. (Tokyo, Japan), was used in this retrospective cohort study. The datasets generated and analyzed during the present study are available from the corresponding author upon reasonable request.

## Supplementary material

Supplementary Figure 1 Changes in Alb, CRP, and lymphocyte counts from admission to day 28 Alb, albumin; CRP, C-reactive protein.
