# Supplementary figures and images for: Intravenous immunoglobulin for mortality and inflammatory status in patients with sepsis: a retrospective database study
Source: Front Immunol. 2025 Jan 16;15:1511481. doi: 10.3389/fimmu.2024.1511481 (PMC11779611; doi:10.3389/fimmu.2024.1511481)

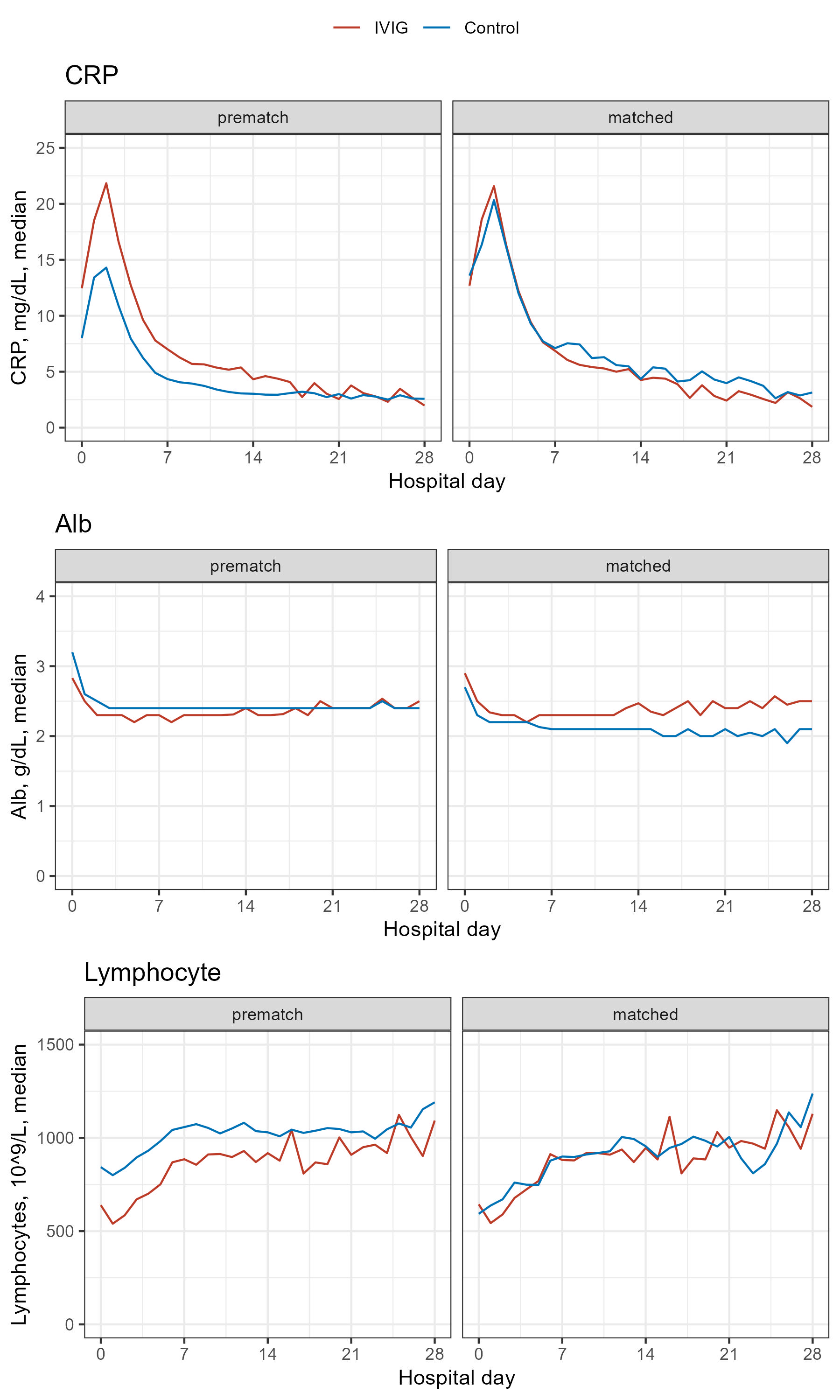

Supplement: Supplementary Figure 1 — Changes in Alb, CRP, and lymphocyte counts from admission to day 28 Alb, albumin; CRP, C-reactive protein. [file Image1.jpeg]
