# Supplementary material for: Intravenous immunoglobulin for mortality and inflammatory status in patients with sepsis: a retrospective database study
Source: Front Immunol. 2025 Jan 16;15:1511481. doi: 10.3389/fimmu.2024.1511481 (PMC11779611; doi:10.3389/fimmu.2024.1511481)
Supplement: Supplementary file 2 [file Table1.docx]

Supplementary Table 1. Patient characteristics before applying the imputation method and the number (percentages) of missing values for each variable.

| Variables | IVIG  (n = 786) | Control  (n = 14,373) |  |
| --- | --- | --- | --- |
| Body mass index, median (IQR) | 22.2 (19.6–24.6) | 21.7 (19.0–24.6) |  |
| Missing, n (%) | 80 (10.2) | 948 (6.6) |  |
| Smoking status, n (%) |  |  |  |
| Current/ex-smoker | 223 (38.6) | 4,973 (38.8) |  |
| Non-smoker | 354 (45.0) | 7,846 (54.6) |  |
| Missing, n (%) | 209 (26.6) | 1,554 (10.8) |  |
| Ambulance use, n (%) | 509 (64.8) | 8,570 (59.8) |  |
| Missing, n (%) | 1 (0.1) | 46 (0.3) |  |
| Laboratory data |  |  |  |
| White blood cell count, median (IQR), 10^9^/L | 7,800 (3,300–12,900) | 9,400 (6,500–13,300) |  |
| Missing, n (%) | | 74 (9.4) | 1,722 (12.0) |
| Lymphocyte count, median (IQR), 10^9^/L | 450 (260–760) | 700 (430–1,090) |  |
| Missing, n (%) | 256 (32.6) | 7,597 (52.9) |  |
| Hemoglobin, median (IQR), 10^9^/L | | 10.4 (9.0–12.0) | 11.2 (9.6–12.8) |
| Missing, n (%) | | 75 (9.5) | 1,724 (12.0) |
| Platelet count, median (IQR), 10^9^/L | | 124 (71–186) | 170 (119–230) |
| Missing, n (%) | | 76 (9.7) | 1,724 (12.0) |
| Total bilirubin, median (IQR), mg/dL | 0.7 (0.5–1.2) | 0.7 (0.5–1.2) |  |
| Missing, n (%) | 92 (11.7) | 2,306 (16.0) |  |
| Creatinine, median (IQR), mg/dL | 1.0 (0.7–1.9) | 0.9 (0.7–1.4) |  |
| Missing, n (%) | 75 (9.5) | 1,739 (12.1) |  |
| Albumin, median (IQR), g/dL | 2.4 (2.0–2.7) | 2.8 (2.3–3.3) |  |
| Missing, n (%) | 83 (10.6) | 2,738 (19.0) |  |
| C-reactive protein, median (IQR), mg/dL | 11.9 (3.3–21.3) | 7.6 (2.1–16.3) |  |
| Missing, n (%) | 83 (10.6) | 2,367 (16.5) |  |

Only variables with missing values are displayed.

Abbreviations; IVIG, intravenous immunoglobulin; IQR, interquartile range.

Supplementary Table 2. Absolute risk differences in outcomes between the IVIG group and control group using the overlap weighting method.

| Outcomes | Absolute risk difference^†^ |
| --- | --- |
| Primary outcome |  |
| 28-day mortality, n (%) | -2.4 (-4.8 to 0) |
| Secondary outcome |  |
| In-hospital mortality, n (%) | -2.5 (-5.3 to -0.3) |

Abbreviations; IVIG, intravenous immunoglobulin.

^†^ Absolute risk differences were presented as percentages with the 95% confidence interval.
